# Kat-ARC accelerated 4D flow CMR: clinical validation for transvalvular flow and peak velocity assessment

**DOI:** 10.1186/s41747-022-00299-5

**Published:** 2022-09-22

**Authors:** Hosamadin Assadi, Bhalraam Uthayachandran, Rui Li, James Wardley, Tha H. Nyi, Ciaran Grafton-Clarke, Andrew J. Swift, Ana Beatriz Solana, Jean-Paul Aben, Kurian Thampi, David Hewson, Chris Sawh, Richard Greenwood, Marina Hughes, Bahman Kasmai, Liang Zhong, Marcus Flather, Vassilios S. Vassiliou, Pankaj Garg

**Affiliations:** 1 University of East Anglia, Norwich Medical School, Norfolk, UK; 2 Norfolk and Norwich University Hospitals NHS Foundation Trust, Norfolk, UK; 3 Division of Molecular and Clinical Medicine, University of Dundee, Dundee, UK; 4 Department of Infection, Immunity and Cardiovascular disease, University of Sheffield Medical School and Sheffield Teaching Hospitals NHS Trust, Sheffield, UK; 5 ASL Europe, GE Healthcare, Munich, Germany; 6 Pie Medical Imaging BV, Maastricht, the Netherlands; 7 National Heart Centre Singapore, 5 Hospital Drive, Singapore, Singapore; 8 Duke-NUS Medical School, 8 College Road, Singapore, Singapore

**Keywords:** Aortic valve, Blood flow velocity, Echocardiography (Doppler), Magnetic resonance imaging, Mitral valve

## Abstract

**Background:**

To validate the k-adaptive-t autocalibrating reconstruction for Cartesian sampling (kat-ARC), an exclusive sparse reconstruction technique for four-dimensional (4D) flow cardiac magnetic resonance (CMR) using conservation of mass principle applied to transvalvular flow.

**Methods:**

This observational retrospective study (2020/21-075) was approved by the local ethics committee at the University of East Anglia. Consent was waived. Thirty-five patients who had a clinical CMR scan were included. CMR protocol included cine and 4D flow using Kat-ARC acceleration factor 6. No respiratory navigation was applied. For validation, the agreement between mitral net flow (MNF) and the aortic net flow (ANF) was investigated. Additionally, we checked the agreement between peak aortic valve velocity derived by 4D flow and that derived by continuous-wave Doppler echocardiography in 20 patients.

**Results:**

The median age of our patient population was 63 years (interquartile range [IQR] 54–73), and 18/35 (51%) were male. Seventeen (49%) patients had mitral regurgitation, and seven (20%) patients had aortic regurgitation. Mean acquisition time was 8 ± 4 min. MNF and ANF were comparable: 60 mL (51−78) *versus* 63 mL (57−77), *p =* 0.310). There was an association between MNF and ANF (rho = 0.58, *p* < 0.001). Peak aortic valve velocity by Doppler and 4D flow were comparable (1.40 m/s, [1.30−1.75] *versus* 1.46 m/s [1.25−2.11], *p* = 0.602) and also correlated with each other (rho = 0.77, *p* < 0.001).

**Conclusions:**

Kat-ARC accelerated 4D flow CMR quantified transvalvular flow in accordance with the conservation of mass principle and is primed for clinical translation.

## Key points

- k-adaptive-t autocalibrating reconstruction for Cartesian sampling (Kat-ARC) is a spatiotemporal-correlation-based autocalibrating parallel imaging method with cardiac motion adaptive temporal window selection.
- Using Kat-ARC in 35 patients, the mitral net flow was in agreement with the aortic net flow.
- In 20 patients, peak velocity at Kat-ARC 4D flow and Doppler echocardiography were comparable and significantly correlated.

## Background

Four-dimensional (4D) flow cardiovascular magnetic resonance (CMR) is emerging as the reference standard for intracardiac flow imaging [1–6]. 4D flow CMR reduces assumptions made by several standard flow imaging methods and allows valve motion to be factored in to measure transvalvular flow more accurately [7–9]. This is particularly important for the precise assessment of valvular heart disease [10].

When compared to two-dimensional (2D) phase-contrast acquisition, 4D flow offers better visualisation of flow in the whole heart and great vessels. Also, it allows us to generate reformatted flow plane in the region of interest after scans [11–14]. This allows flexibility to explore flow patterns in more detail without the requirement of patient being in the scanner all the time. Especially in congenital heart disease, where composite flow is calculated in several planes through complex vascular associations, 4D flow allows the patient to be scanned on a non-clinically supervised list with full coverage of the chest [1] In addition to flow quantification, 4D flow can be used to assess novel emerging haemodynamic parameters [15–17].

However, 4D flow has had issues with long scanning time and several magnetic resonance imaging (MRI) vendors are progressively updating their 4D flow sequences for faster and accelerated imaging to save time. Several imaging acceleration methods on magnetic resonance systems from a variety of vendors have been validated for this purpose [18–23]. The transvalvular flow quantification, particularly for mitral and tricuspid valves, is retrospectively gated to avoid temporal blurring [10].

MRI hardware vendors have made significant iterative development in 4D flow sequences. One such 4D flow sequence uses both parallel imaging and compressed sensing acceleration called k-adaptive-t-autocalibrating reconstruction for Cartesian sampling (Kat-ARC). Previous versions of this sequence, using L1-SPIRiT, have been tested for both inlet and outlet flow quantifications [20, 24] or shunt evaluation [25, 26]. However, the current iterative 4D flow sequence has not been externally validated using commercially available 4D flow post-processing software solutions. This is an important step in establishing the validity and clinical translation of the available 4D flow sequence.

The main objective of this research study was to clinically validate Kat-ARC4D flow CMR for transvalvular flow quantification using the conservation of mass principle. In addition, in a subcohort of patients where transthoracic echocardiography (TTE) data was available, we aimed to investigate the agreement of aortic valve peak velocity between TTE and 4D flow CMR.

## Methods

### Study cohort

For this study, we retrospectively included 35 cases from our routine CMR service. Inclusion criteria were baseline functional cine images and 4D flow CMR assessment. Only patients who were outpatients and clinically stable were recruited. The exclusion criteria were limited to any MRI contraindications.

### Ethics approval

This study was approved by the local ethics committee at the University of East Anglia as an observational retrospective study (2020/21-075). Consent was waived. The study complied with the Declaration of Helsinki.

### CMR protocol

CMR studies were conducted on a 3-T Discovery MR750w GE system (GE Healthcare, Milwaukee, WI, USA), equipped with an 8-channel HD cardiac Array coil. The protocol included baseline survey images and 30-phase cine sequences. Cine images were acquired during end-expiratory breath-hold with an electrocardiographically gated 2D fast imaging employing steady-state acquisition (FIESTA) single-slice breath-hold sequence. Long-axis electrocardiographically gated 2D FIESTA cine in four-chamber, three-chamber, and two-chamber planes and short-axis electrocardiographically gated 2D FIESTA cine images were also acquired. The number of left ventricular (LV) short-axis slices was dependent on the size of each patient’s heart. LV short-axis images were post-processed to calculate functional status as per standard techniques.

### 4D flow CMR acquisition

**Fig. 1:**
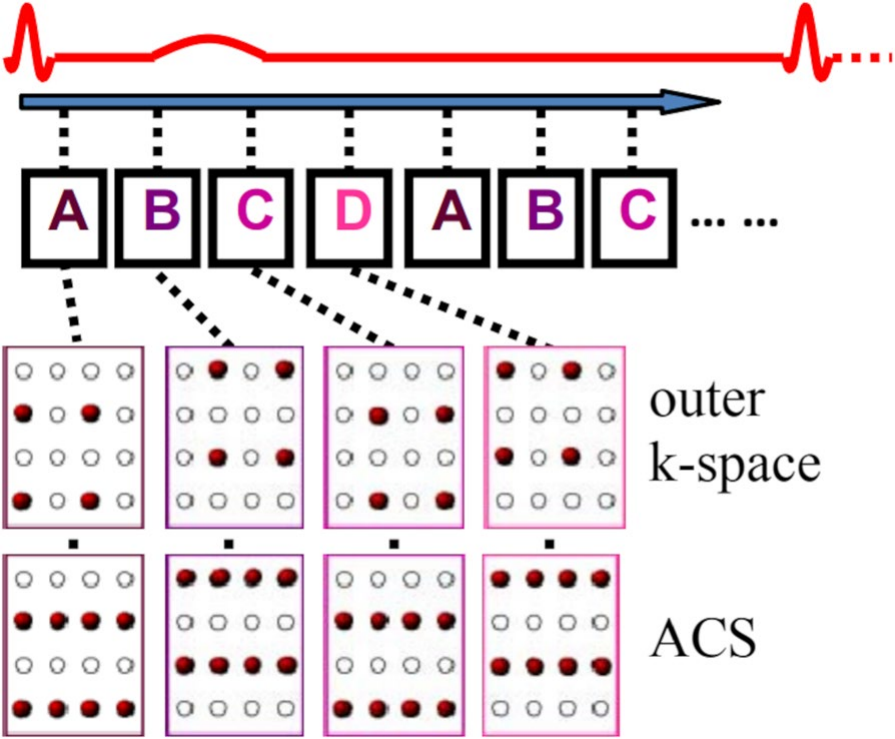
A scheme of the k-space acquisition using the HyperKat/Kat-ARC sequence. *ACS* Autocalibration signal

The initial velocity encoding (VENC) setting for 4D flow CMR was 150−400 cm/s for all cases. This was optimised depending on previously available echocardiography data. If there was no history of valvular heart disease, then we choose a VENC of 150 cm/s. In the context of known valvular heart disease, we choose the VENC at the maximum velocity recorded during echocardiography assessment. Table 1 describes the technical details of the 4D flow Kat-ARC (or HyperKat), a spatiotemporal-correlation-based autocalibrating parallel imaging method with cardiac motion adaptive temporal window selection [27]. The k-t sampling scheme used variable density to improve accuracy and reduce coherent residual artefacts (Fig. 1). Additionally, a static tissue removal scheme was used to identify voxels with no flow or motion and remove the signal from such static voxels prior to Hyperkat processing. This reduces residual aliasing artefacts at their high acceleration during the reconstruction. Field-of-view was planned to cover the whole heart, aortic valve, and proximal ascending aorta only. HyperKat acceleration with a factor of 6 was used. Other standard scan parameters were as follows: field-of-view 340 mm × 340 mm; acquired voxel size 3 × 3 × 3 mm^3^ and reconstructed voxel size 1.5 × 1.5 × 1.5 mm^3^. The number of phases was kept consistent to cine sequences at 30 cardiac phases.

**Table 1:** Technical parameters of the four-dimensional flow sequence

Acceleration method	HyperKat factor 6 with compressed sensing
Flip angle (degrees)	8
Velocity encoding (cm/s)	150
Field of view (mm)	350–400
Slice thickness (mm)	3
Echo time (ms)	2.14
Repetition time (ms)	4
Number of excitations	4
Electrocardiographic gating	Retrospective
Respiratory compensation	Free-breathing
Acquisition temporal resolution (ms)	48
Reconstructed number of phases	30
Spatial resolution, acquired (mm^3^)	3 × 3 × 3 (isotropic)

### 4D flow CMR analysis

Post-processing was done on a commercially available software package (CAAS MR, version 5.1, Pie Medical Imaging, Maastricht, The Netherlands). Automated velocity offset corrections were applied. Automated valve tracking was done for two orthogonal views of the mitral and aortic valves. If necessary, the valve plans were corrected for some cardiac phases. The automated region of interest contours on the reformatted planes was manually corrected for both systolic and diastolic phases. The following was recorded: mitral forward flow (MFF), mitral backward flow (MBF), aortic forward flow (AFF), and aortic backward flow (ABF). Aortic net flow was calculated as AFF minus ABF. Mitral net flow was calculated as MFF minus MBF. For the peak velocity assessment, we used a prototype software solution from Pie Medical Imaging (CAAS MR, version 5.2), which automatically traces the peak velocity of the flow streamlines emitted from the dynamic valve plane.

### Echocardiography

**Fig. 2:**
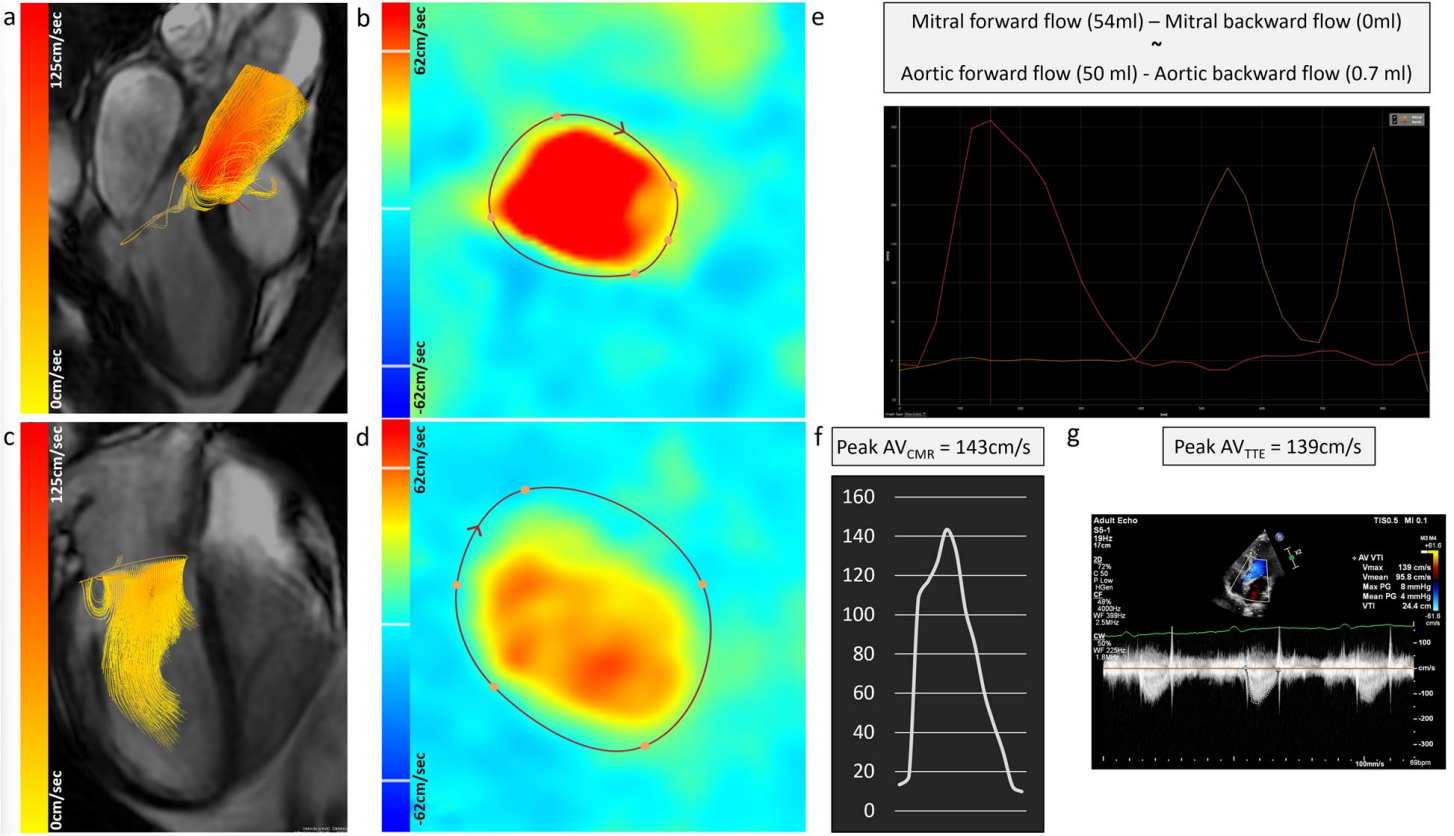
A case example from the study. Flow streamlines of aortic forward flow (**a**). Quantification of aortic forward flow using the reformatted phase-contrast plane (**b**). Mitral inflow quantification using retrospective valve tracking procedures (**c**, **d**). Demonstration of total flow and conservation of mass principle, *i.e.*, mitral forward flow (MFF) minus mitral backward flow (MBF) is equal to aortic forward flow (AFF) minus aortic backward flow ABF (**e**). Peak velocity through the aortic valve using three-dimensional streamlines to automatically trace the transvalvular peak (**f**). Echocardiography continuous Doppler method for peak velocity assessment through the aortic valve (**g**)

All echocardiograms were performed according to the British Society of Echocardiography guidelines for TTE examination [28], using 3 x GE E95, 4 x Philips Epiq 7, and 1 X Philips CX50. Continuous-wave Doppler TTE was used to measure the peak velocity through the aortic valve in apical 3-chamber views (Fig. 2).

### Statistical analysis

Test for normal distribution was done using Shapiro-Wilk test. Due to non-normal distribution, continuous variables were reported as median and interquartile range (IQR). As flow data was non-parametric, we have used non-parametric statistics. Wilcoxon paired *t* test was performed to compare the difference between the different flows. Correlations were evaluated using Spearman's coefficient of rank correlation (rho). Bland-Altman plots were constructed to assess the agreement between methods. A *p*-value of less than .05 was deemed to be statistically significant. Data analyses were performed using MedCalc® Statistical Software version 20.011 (MedCalc Software Ltd., Ostend, Belgium).

## Results

The mean acquisition time for 4D flow CMR using the Kat-ARC sequence was 8 ± 4 min. The median age of our patient population was 63 years (IQR 57−77), and 18 (51%) were males. Seventeen (49%) patients had mitral regurgitation (MR), and 7 (20%) patients had aortic regurgitation (AR). The demographic data for all 35 patients are detailed in Table 2.

**Table 2:** Patient characteristics (*n* = 35)

Clinical variable	Values
Age (years) (median, IQR)	63 (57−77)
Height (cm) (median, IQR)	169 (163−177)
Weight (kg)	80 (68−92)
Male gender (%)	18 (51)
Hypertension (%)	13 (37)
Diabetes mellitus (%)	3 (9)
Hypercholesterolaemia (%)	6 (17)
Ischaemic heart disease (%)	9 (26)
Mitral regurgitation (%)	17 (49)
Aortic regurgitation (%)	7 (20)

*IQR* Interquartile range

Median LV end-diastolic volume was 132 mL (IQR 112−187) and LV end-systolic volume was 48 mL (IQR 33−76). On Kat-ARC 4D flow CMR, median MNF and ANF were comparable: 60 mL (51−78) *versus* 63 mL (57−77), *p =* 0.310). The median aortic valve forward and backward flow measurements were 70 mL/s (IQR 59−77) and 0.2 mL/s (IQR 0−3.5), respectively. Moreover, 4D flow CMR-derived MFF and MBF for these patients were 71 mL/s (IQR 58−90) and 8 mL/s (IQR 4−13), respectively. Median Doppler’s peak aortic valve velocity and 4D flow CMR derived peak aortic valve velocity were comparable with no significant differences (1.40 m/s, [1.30−1.75] *versus* 1.46 m/s [1.25−2.11], *p* = 0.602). The CMR study findings are summarised in Table 3.

**Table 3:** Cardiovascular magnetic resonance (CMR) functional/flow quantification and echocardiographic peak aortic valve velocity

CMR Cine functional parameters	Median (IQR)
LV end-diastolic volume (mL)	132 (112−187)
LV end-systolic volume (mL)	48 (33−76)
LV stroke volume (mL)	89 (72−106)
LV mass (g)	135 (115−186)
LV ejection fraction (%)	64 (51−69)
**CMR four-dimensional flow parameters**	
Mitral valve forward flow (mL)	71 (58−90)
Mitral valve backward flow (mL)	8 (4−13)
Aortic valve forward flow (mL)	70 (59−77)
Aortic valve backward flow (mL)	0.20 (0−3.5)
Aortic net flow (mL)	63 (57−77)
Mitral net flow (mL)	60 (51−78)
Peak Aortic valve velocity (m/s)	1.46 (1.25−2.11)
**Echocardiographic peak aortic valve velocity (m/s)**	1.40 (1.30−1.75)

*IQR* Interquartile range, *LV* Left ventricle

In a subcohort of 20 patients with echocardiography data (12 males, 60%; median age 62 years (IQR 59−76), their median LV ejection fraction, mass and stroke volume were 66 mL (IQR 53−73), 140 mL (IQR 114−190), and 88 mL (IQR 72−105), respectively. Moreover, median LV end-diastolic volume was 129 mL (IQR 111−154) and median LV end-systolic volume was 45 mL (IQR 30−70).

**Fig. 3:**
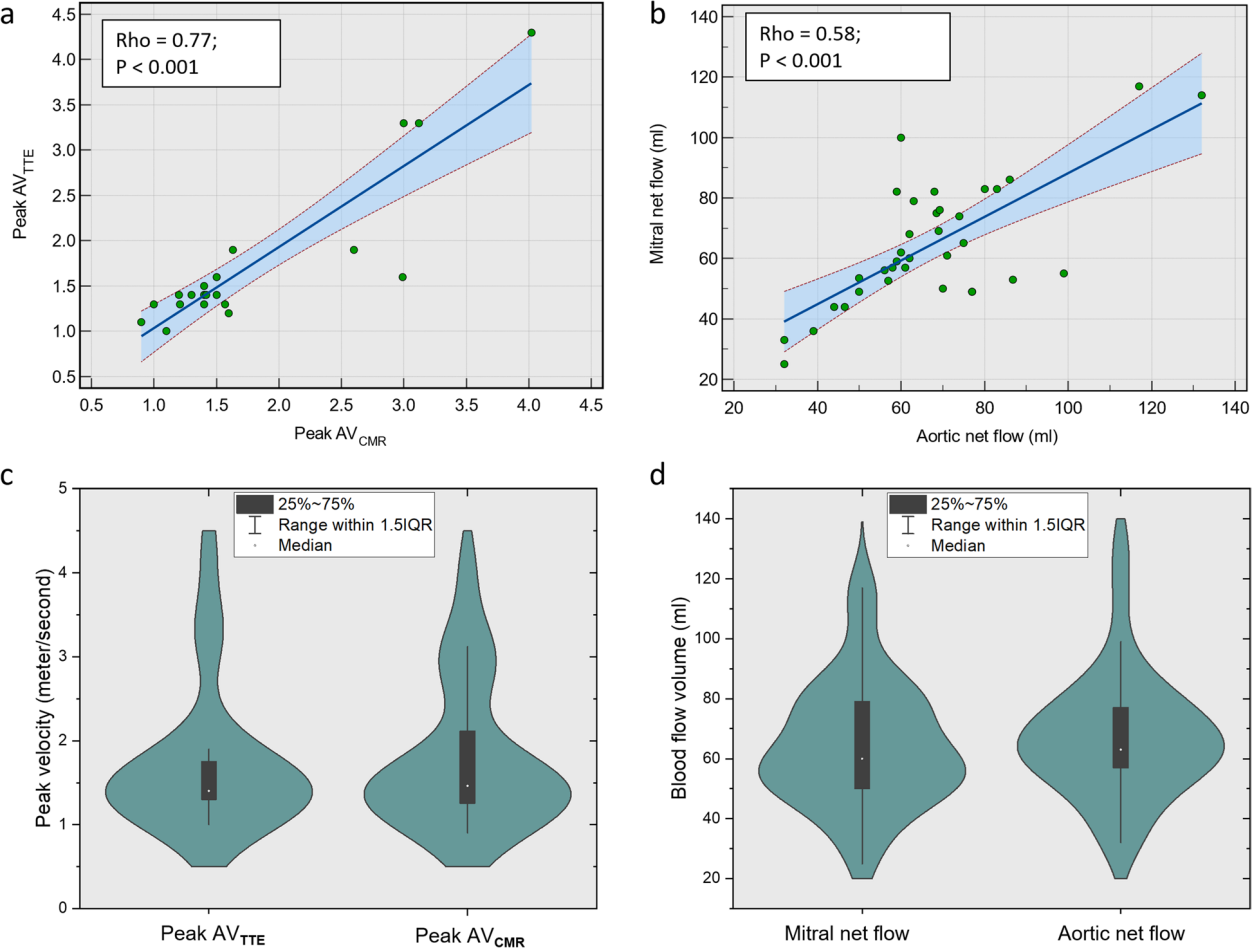
**a**, **b** Scatter plots with 95% confidence interval demonstrating a correlation between mitral and aortic flows and peak velocity through the aortic valve by echocardiography and four-dimensional flow cardiovascular magnetic resonance (**a**, **b**). Violin-plot showing minimal differences between each group (**c**, **d**)

As shown in Fig. 3, there was a significant association between MNF and ANF (rho = 0.58, *p* < 0.001). Moreover, a significant positive correlation was observed between peak aortic velocity by Doppler and 4D flow CMR (rho = 0.77, *p* < 0.001). Both groups demonstrated minimal differences on violin-plots when compared to each other.

**Fig. 4:**
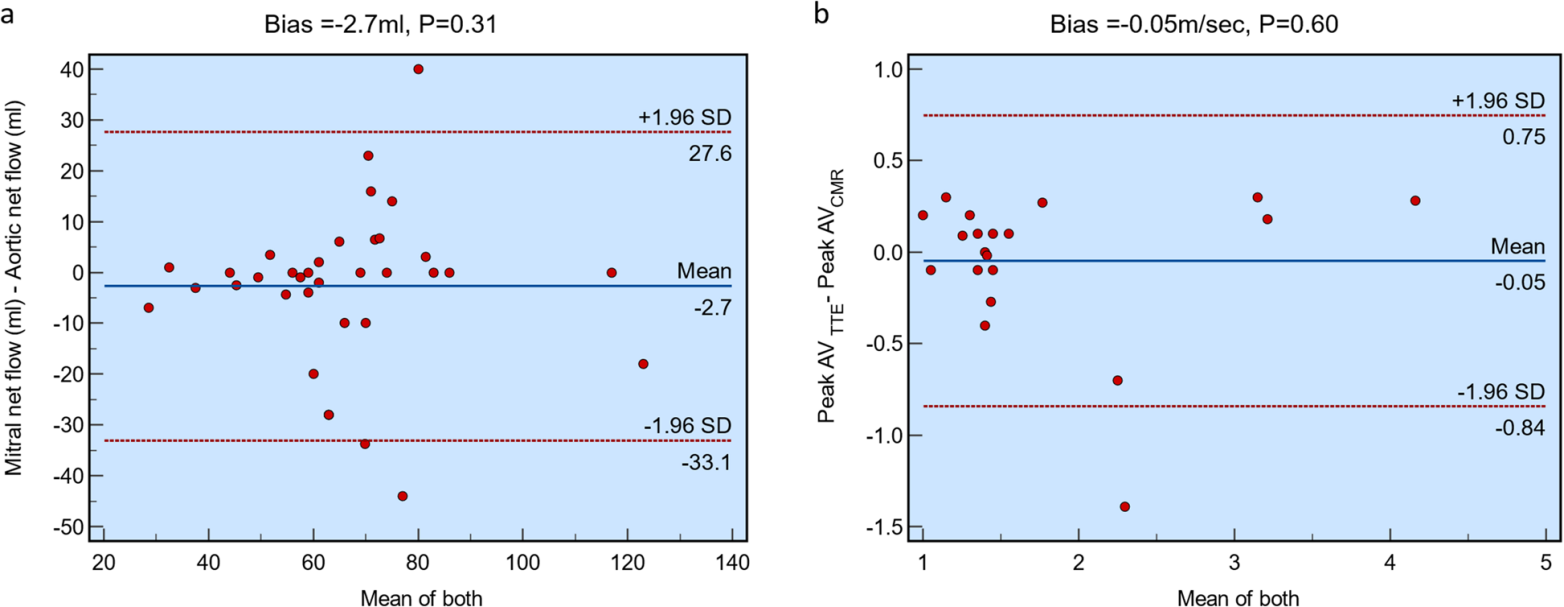
Bland-Altman plots demonstrating no significant bias between each group

In Fig. 4, Bland-Altman analysis used to assess the agreement between the net aortic/mitral flows and the peak velocity through the aortic valve using echocardiography and 4D flow CMR is shown. No significant biases were observed between groups (-2.7 mL, *p* = 0.310) and (-0.05 m/s, *p* = 0.603), respectively.

## Discussion

In this study, we aimed to test the Kat-ARC sequence for 4D flow imaging to quantify transvalvular flow using a commercially available software solution. The first result was a good agreement in mitral and aortic transvalvular flow as evaluated by this sequence. In addition, the peak velocity assessment through the aortic valve was in agreement with continuous-wave Doppler echocardiography. The findings of this study play in favour of a broader adoption of the Kat-ARC 4D flow sequence for valvular heart disease assessment.

Our group has previously validated echo-planar imaging (EPI) accelerated 4D flow sequence (acceleration factor 5) using similar conservation of mass principle [18]. The acquisition time by both EPI and Kat-ARC sequences are similar, roughly 8 min. Similar to EPI acceleration, all image quality using the Kat-ARC 4D flow sequence was adequate for postprocessing and quantification. However, EPI underestimates peak velocity, especially when the flow is parallel to the readout or blip phase-encoding gradient. Using the Kat-ARC 4D flow sequence, we did not observe any underestimation of peak velocity compared to Doppler methods. Notably, several studies have now established that respiratory navigation is not essential for intra-cardiac flow quantification using 4D flow CMR [18, 19, 22], especially in the adult population. This study builds on that evidence and gives similar results. Non-respiratory navigated 4D flow almost halves acquisition time, making it more clinically feasible in routine practice.

One of the relevant differences in previous studies and the current work is that this work solely involves patients with possible valvular heart disease in routine clinical practice. The conservation of mass principle in patients with mitral regurgitation and aortic regurgitation was observed in this study. This builds confidence in 4D flow CMR in a real-world setting to assess valvar heart disease. In addition, this study uniquely compares echocardiography acquired peak velocity through the aortic valve with 4D flow CMR derived peak velocity. A study by Hälvä et al. [29] debates the reliability of 4D flow for peak velocity assessment. However, their work used prospectively gated 4D flow sequences, which have the issue of temporal blurring. Importantly, it is noteworthy that Doppler echocardiography is by no means a ‘reference standard’ and can overestimate the peak velocity, as shown by a previous work, against the invasive assessment reference, demonstrated that Doppler overestimates peak velocity, and 4D flow CMR was consistent with invasively acquitted peak pressure drop [30]. 4D flow application beyond cardiology have been demonstrated and has shown promising results in the assessment of abdominal haemodynamics such as changes in flow and vessel morphology [31, 32], risk stratification [33] and presurgical planning, and follow-up [34].

We acknowledge this study has limitations. Firstly, this study mainly quantified flow for the left-sided valves, notably because the data did not include right-heart multiplanar cine sequences. Secondly, we did not carry out any *ex vivo* phantom experiments to check the robustness of the sequence. However, the 4D flow sequence was tested in a clinical environment, which is arguably the strength of this work. This study is also limited to one centre and one MRI field strength (3 T). In addition, we did not quantify right-heart flows as we had limited right-heart cine sequences to do robust valve tracking and we did not compare Kat-ARC with other 4D flow sequences. Finally, this study does not represent patients with arrhythmias in whom temporal blurring may happen due to arrhythmia rejection algorithms. Hence, the findings of this study should not be applied in patients with significant arrhythmias.

In conclusion, we showed that Kat-ARC accelerated 4D flow CMR enables to quantify transvalvular flow according to the conservation of mass principle and is primed for clinical translation.

## Data Availability

This study uses patient data and, as such, is not available. However, some post-process and fully anonymised data can be made available at the discretion of the corresponding author.

## Abbreviations

2D: Two-dimensional
4D: Four-dimensional
ABF: Aortic backward flow
AFF: Aortic forward flow
ANF: Aortic net flow
CMR: Cardiovascular magnetic resonance
EPI: Echo-planar imaging
FIESTA: Fast imaging employing steady-state acquisition
IQR: Interquartile range
Kat-ARC: k-adaptive-t autocalibrating reconstruction for Cartesian sampling
LV: Left ventricular
MBF: Mitral backward flow
MFF: Mitral forward flow
MRI: Magnetic resonance imaging
TTE: Transthoracic echocardiography
VENC: Velocity encoding
